# Rapid Enhanced MM3-COPRO ELISA for Detection of *Fasciola* Coproantigens

**DOI:** 10.1371/journal.pntd.0004872

**Published:** 2016-07-20

**Authors:** Victoria Martínez-Sernández, Ricardo A. Orbegozo-Medina, Marta González-Warleta, Mercedes Mezo, Florencio M. Ubeira

**Affiliations:** 1 Laboratorio de Parasitología, Facultad de Farmacia, Universidad de Santiago de Compostela, Santiago de Compostela, Spain; 2 Laboratorio de Parasitología, Centro de Investigaciones Agrarias de Mabegondo, INGACAL, Abegondo, A Coruña, Spain; McGill University, CANADA

## Abstract

ELISA-based methods of detecting *Fasciola* cathepsins in feces are powerful techniques for diagnosing infections by *F*. *hepatica* and *F*. *gigantica*. In the last decade, the in-house MM3-COPRO ELISA and its commercial version BIO K 201 (BIO X Diagnostics, Belgium) have been recognized as useful tools for detecting early infections by such trematodes and for monitoring the efficacy of anthelmintic treatments in human and animal species, as they provide some advantages over classic fecal egg counts. However, the sensitivity of MM3-COPRO ELISA can sometimes be compromised by the high variability in the concentration of cathepsins in fecal samples throughout the biological cycle of *Fasciola* (mainly in cattle) and by differences in the between-batch performance of peroxidase-labeled anti-mouse IgG polyclonal antibodies. To prevent such problems, we investigated whether the incorporation of a commercial streptavidin-polymerized horseradish peroxidase conjugate, in order to reveal bound biotinylated monoclonal antibody MM3, can improve the sensitivity of the MM3-COPRO ELISA. We observed that inclusion of this reagent shifted the previous detection limit of the assay from 0.6 ng/mL to 150 pg/mL and that the modified test is able to identify infection in cows harboring only one fluke. Moreover, we demonstrated that maximal OD values can be achieved with short incubations (30 min each step) at RT with shaking, rather than standard incubations, which significantly accelerates the diagnostic procedure. Finally, we did not find a significant correlation between coproantigen concentration and parasite burden in cattle, which may be due to the low parasite burden (1–10 adult flukes) of the animals used in the present study. As the usefulness of the classic MM3-COPRO test for detecting animal and human infections has already been demonstrated, it is expected that the improvements reported in this study will add new insights into the diagnosis and control of fasciolosis.

## Author Summary

We have previously reported how the combined use of mAb MM3 with polyclonal antibodies obtained from rabbit immunized with *Fasciola hepatica* excretory-secretory antigens led to the development of the in-house MM3-COPRO ELISA and its commercial version BIO K 201 (BIO X Diagnostics, Belgium), which are widely used to detect human and animal infections caused by *F*. *hepatica*. After more than a decade in use, both tests have proven to be useful tools for specifically detecting *Fasciola* infections, although it has also been found that: i) the conditions of use of the commercial test in some field studies did not enable the sensitivity obtained with the in-house test to be reached, and ii) the batches of the secondary reagent (peroxidase-labeled anti-mouse antibodies) currently available for use in the in-house test do not perform the same as previous batches. To solve these problems, we provide data showing that the incorporation of an enhancement system consisting of streptavidin-polymerized horseradish peroxidase conjugate greatly improved the sensitivity of the MM3-COPRO ELISA and enabled reduction of the incubation time. These modifications enabled the detectability of the assay to be 150 pg/mL, thus enabling detection of infection in animals harboring only one fluke.

## Introduction

Fascioliasis (= fasciolosis) is a worldwide emergent zoonotic disease produced by infection with trematodes of the genus *Fasciola*. The two main species of this genus, *F*. *hepatica* and *F*. *gigantica*, are pathogenic to humans and livestock [1]. Considering the worldwide distribution, *F*. *gigantica* is the only species present in Western Africa, while *F*. *hepatica* is the only species present in Europe, the Americas, Australia and the African Magreb [2]. However, both species have been reported to coexist in Eastern and Southern Africa as well as in several regions of Asia [3]. The existence of two species with overlapping regions has implications for developing sensitive diagnostic tests of general application.

Typically, diagnosis of human and animal infections caused by *Fasciola* species is carried out by coproscopy or immunological techniques, including determination of circulating antigens in serum, measurement of coproantigens and detection of serum antibodies [4, 5]. Although coprological techniques are advantageous in terms of the cheapness of laboratory material and detection of active infections, they are time-consuming, require expert personnel and have poor sensitivity. Serological methods have the advantage of permitting easy automation, which is of great interest for handling large volume of samples. These methods are also very sensitive and can be used for early monitoring of *Fasciola* infections in herds by using either serum or milk samples [6]. However, these techniques do not differentiate between antibodies induced by current infections/reinfections and those still present in animals or humans successfully treated with anthelmintics during the course of a past *Fasciola* infection. Methods for detecting circulating *Fasciola* antigens and/or coproantigens solve the above mentioned problems associated with coprological and serological techniques. However, detection of coproantigens is preferred as sampling is not invasive and the presence of antigens in feces is not limited by time, as may occur with circulating antigens. Moreover, these methods are of widespread application, as the same techniques can be used to detect *Fasciola* coproantigens in fecal samples from humans and animal species.

In the past decades, several capture ELISA methods that use monoclonal and/or polyclonal antibodies were reported to be able to detect small amounts of specific *Fasciola* coproantigens in fecal samples [7–10]. However, since then, only the BIO K 201 kit (BIO X Diagnostics, Belgium), i.e. the commercial version of MM3-COPRO ELISA [9], has been globally used. Since being commercialized (in 2007), both versions of the test have been recognized as valuable diagnostic tools for detecting *Fasciola* infections or for monitoring the efficacy of treatment with anthelmintics in several studies [9–16]. Nevertheless, during years of use of the MM3-ELISA tests, two drawbacks have also been highlighted: i) the difficulty with the commercial test [17–20], which uses avidin-horseradish peroxidase (HRP) as a secondary conjugate, in maintaining the sensitivity of the original in-house MM3-COPRO test, and ii) the dependence of the in-house MM3-COPRO test on particular batches of HRP-labeled anti-mouse IgG antibodies, for yielding good sensitivity and specificity. To prevent these disadvantages and to guarantee a homogeneous and highly sensitive product, we investigated the possibility of substituting the above secondary reagents with a streptavidin (SA)-polymerized HRP (PolyHRP) detection system and evaluated the conditions of use of this reagent. We found that this ELISA enhancement system is an improvement on classic secondary reagents as it increases the sensitivity of the above ELISA tests while enabling a reduction in the incubation time required.

## Materials and Methods

### Ethics statement

The biological samples used in the present study were of animal origin (sheep and cattle) and were obtained from a collection of frozen fecal samples stored at INGACAL (Mabegondo, A Coruña, Spain). The samples were obtained during routine diagnostic procedures and from experimental infections with *F*. *hepatica* reported in previous studies [9, 21, 22].

### *Fasciola hepatica* excretory-secretory antigens

*Fasciola* excretory-secretory antigens (ESAs) were obtained as previously described [23, 24]. Briefly, live adult flukes collected from bile ducts of naturally infected cows were washed, first in sterile saline and glucose (2 g/L) at 38°C and then in RPMI 1640 cell culture medium supplemented with 20 mM HEPES, 0.3 g/L L-glutamine, 2 g/L sodium bicarbonate and antibiotics (penicillin and streptomycin) at 38°C under 5% CO_2_ in air. The flukes were then transferred to 75-cm^2^ tissue culture flasks and maintained in culture medium (3 mL/fluke) at 38°C under 5% CO_2_ in air. After incubation for 24 h, the medium containing ESAs was removed and centrifuged at 10,000 *g* for 20 min at 4°C in the presence of protease inhibitors (SigmaFast Protease Inhibitor Tablets; Sigma-Aldrich, Madrid, Spain). The supernatant was passed through a 0.45 μm pore filter disk and concentrated in an Amicon 8050 ultrafiltration cell (Amicon, Inc., Beverly, MA) equipped with a YM10 membrane (10 kDa cut-off), dialyzed against PBS, sterilized by filtration, and stored at -80°C until required. Protein concentration was measured using the Pierce BCA Protein Assay Kit (Thermo Fisher Scientific, Barcelona, Spain).

### Fecal samples

#### Cattle

Positive and negative fecal samples were collected in the slaughterhouse from respectively adult cattle (Friesian and ‘Rubia Gallega’ breed) naturally infected with *F*. *hepatica* and cattle free of *F*. *hepatica* (S1 Fig). After the animals were slaughtered, the livers were examined, and all flukes were collected and counted (gold standard of infection). The absence of *F*. *hepatica* infection in negative cattle was also confirmed serologically by the MM3-SERO test [21]. All fecal samples were analyzed microscopically to detect the presence of eggs by a quantitative sedimentation technique [25], and tested for the presence of *Fasciola* coproantigens using the classic MM3-COPRO ELISA test (hereafter referred to as model A) [9] before the samples were stored frozen until analysis by other variants of the test evaluated (models B, C and D). For comparison between the different MM3-COPRO ELISA models evaluated in the present study, only samples from animals with low parasite burden (1–10 flukes; n = 18) were investigated. Also, among the negative cattle (n = 30), some were naturally infected with *Calicophoron daubneyi* (counts ranging from 103 to 11,895 flukes; n = 9) and intestinal nematodes were also present in most animals.

For experimental infections, four cows reared under *Fasciola*-free conditions were infected once with 25 (n = 2) and 50 (n = 2) *F*. *hepatica* metacercariae obtained in our laboratory from experimentally infected *Galba truncatula* snails. Fecal samples were obtained before the infection and weekly for 48 weeks postinfection (wpi). At the end of the experiment (48 wpi), one cow was slaughtered and liver parasites collected.

#### Sheep

All sampled sheep were of the Galician autochthonous breed. Negative fecal samples were collected from sheep reared on a *Fasciola*-free farm (n = 18; most of which had intestinal and lung nematodes) and from negative sheep experimentally infected with *Dicrocoelium dentriticum* (n = 2). Positive samples (n = 16) were obtained from 4-month-old lambs reared under *Fasciola*-free conditions and subsequently infected once with 5 (n = 6), 10 (n = 5), 20 (n = 2), or 40 (n = 3) *F*. *hepatica* metacercariae (S1 Fig). Fecal samples were collected immediately before infection and weekly thereafter. All samples were stored frozen at -20°C until use. At 13 wpi, ten randomly chosen animals were sacrificed and necropsied to confirm infection. Among these animals, one was parasitized with 28 flukes and the remaining sheep had 13 or fewer flukes. The remaining sheep were maintained to examine the kinetics of coproantigen release until 18 wpi. Infection of these animals was also confirmed by necropsy.

### Processing fecal samples for ELISA determinations

Fresh or stored (-20°C) individual ovine and bovine stools were mixed with distilled water at a ratio of 1:4 (1 g + 4 mL) or 1:1 (3 g + 3 mL), respectively. The samples were resuspended by mixing on a vortex mixer and centrifuged for 15 min at 1,000 g. The supernatants were collected and subsequently analyzed for the presence of *F*. *hepatica* coproantigens by ELISA.

To evaluate the detectability of the MM3-COPRO test with three secondary reagents, a pool of 6 negative cattle fecal samples were mixed with distilled water, and the supernatant was collected as described previously. The supernatants were used to prepare two-fold dilutions of *F*. *hepatica* ESAs starting at 20 ng/mL prior to ELISA analysis.

### Adjustment of MM3-COPRO ELISA conditions

#### Determination of optimal conditions for use of SA-PolyHRP

ELISA plates were prepared as previously described [10] and used to perform all experiments in the present study. Polystyrene microtiter 1x8 F strip plates (Greiner Bio-One; Soria-Melguizo, Madrid, Spain) were coated overnight (ON) with 100 μL/well of rabbit anti-*Fasciola* polyclonal IgG antibodies (wells in the odd-numbered rows) or polyclonal IgG antibodies from non-immunized rabbits (wells in the even-numbered rows) at 10 μg/mL in PBS. Plates were washed three times with PBS and blocked with 1.5% of sodium caseinate in PBS for 1 h at room temperature (RT).

Two concentrations of *F*. *hepatica* ESAs (5 and 0.62 ng/mL) diluted in CoproGuard [10] were added in quadruplicate (two odd-numbered wells plus two even-numbered wells) and the plates were incubated for 30 min at RT with shaking at 750 rpm on a microtiter plate shaker (orbit diameter: 1.5 mm). The plates were washed 6 times with PBS containing 0.05% Tween 20 (PBS-T) before 100 μL/well of biotinylated monoclonal antibody (mAb) MM3 diluted 1/10,000 in PBS-T containing 1% BSA (fraction V, Sigma-Aldrich; PBS-T-BSA) was added and the plates were incubated for 30 min at RT with shaking at 750 rpm. The plates were washed again and incubated with the SA-PolyHRP conjugate (Pierce, Thermo Fisher Scientific) at different concentrations (1/5,000, 1/6,500, 1/8,000, 1/10,000) in PBS-T-BSA for 30 min at RT with shaking at 750 rpm. After a washing step, the substrate OPD (SigmaFast OPD, Sigma-Aldrich) was added (100 μL/well) and incubated for 20 min at RT, and the reaction was stopped by addition of 3 N H_2_SO_4_ (25 μL/well). Finally, the optical density (OD) was measured at 492 nm. The OD value for each sample was calculated as OD1–OD2, where OD1 is the mean for the 2 odd-numbered wells (coated with anti-*Fasciola* polyclonal antibodies), and OD2 is the mean for the 2 even-numbered wells (coated with irrelevant polyclonal antibodies).

Once the optimal dilution of SA-PolyHRP was selected (1/8,000), an ELISA was conducted under the same conditions with the exception of the antigen incubation step. In this assay, two-fold dilutions of ESAs ranging from 5 ng/mL to 0.15 ng/mL in CoproGuard were incubated for 30 min, 1 h and 2 h at RT with agitation.

For the initial assays, the preservative CoproGuard was used to dilute ESAs because the antigens cannot be optimally detected by MM3-COPRO ELISA if diluted in distilled water. This may be due to lowered affinity between coated antibodies and the recognized antigens under conditions of low ionic strength. However, for subsequent studies using fecal samples, we used distilled water for the extraction because it is widely used and its use enabled comparison with data obtained with the classic MM3-COPRO ELISA (model A), which was performed following the method described by Mezo et al. [9].

#### Performance of three HRP-labeled secondary detection reagents under different incubation conditions

The ELISA test was performed by adding in quadruplicate (2 odd-numbered wells plus 2 even-numbered wells) two-fold dilutions of ESAs starting at 20 ng/mL in CoproGuard and incubating the plates under different conditions: ON at 4°C or 2 h at 37°C (standard incubations), and for 30 min at RT with shaking at 750 rpm (short incubations). Monoclonal antibody MM3 and three different secondary reagents were subsequently added after a washing step with PBS-T. Both incubation steps were performed for 90 min and 1 h, respectively, at 37°C (standard incubations), or for 30 min at RT with shaking at 750 rpm each (short incubations). Biotinylated MM3 (1/10,000) and PBS-T-BSA (as dilution buffer) were used for the NeutrAvidin (NA)-HRP (Pierce, Thermo Fisher Scientific; dilution 1/2,000) and SA-PolyHRP conjugates (dilution 1/8,000). Unlabeled MM3 diluted 1/2,000 and PBS-T containing 1% skimmed dry milk (PBS-T-SM) were used for the HRP-labeled goat anti-mouse IgG secondary antibodies (EIA grade, Bio-Rad, Madrid, Spain; dilution 1/3,000). This conjugate is referred to as the “current batch” throughout the manuscript for comparative studies with a “former batch” of the same commercial source. The plates were then washed, the OPD substrate was added and the OD values were read as described above.

Once the optimal incubation conditions were determined (incubation of 30 min with shaking at 750 rpm for all incubation steps), an ELISA was performed to evaluate the performance of the three detection reagents in the presence of fecal samples. The same dilutions of ESAs were prepared in fecal supernatants obtained from a pool of 6 negative cattle fecal samples and were tested under the selected conditions. In addition, the SA-PolyHRP conjugate was tested with two HRP chromogens: OPD and TMB (TMB microwell peroxidase substrate system; KPL, Inc., MD, USA). After TMB substrate was added (100 μL/well) and incubated for 5 min at RT, the reaction was stopped by addition of an equal volume of 1 M H_3_PO_4_ and the OD values were measured at 450 nm. The OD value for each sample was calculated as mentioned above (by subtracting the mean OD value of wells with irrelevant antibodies from the mean OD value of wells with anti-*Fasciola* antibodies).

### Detection of *F*. *hepatica* coproantigens in ovine and bovine feces

#### ELISA model A

This method corresponds to the classic MM3-COPRO ELISA [9], which was performed at the time of sample collection. Each fecal supernatant was added in quadruplicate (2 odd-numbered wells plus 2 even-numbered wells) and incubated ON at 4°C. The plates were washed 6 times with PBS-T, and mAb MM3 diluted 1/2,000 in PBS-T-SM was added to each well and incubated for 90 min at 37°C. Bound MM3 was detected by incubation, first with a former batch of HRP-labeled goat anti-mouse IgG antibodies (EIA grade, Bio-Rad) diluted 1/3,000 in PBS-T-SM for 1 h at 37°C and then with OPD, after a washing step. The OD value for each sample was calculated as OD1-OD2, where OD1 is the mean value for wells coated with anti-*Fasciola* antibodies and OD2 is the mean value for wells coated with irrelevant antibodies. The same formula was applied for models B, C and D.

#### ELISA models B and C

Each fecal supernatant was added in quadruplicate, as above, and incubated for 30 min at RT with shaking at 750 rpm (short incubations). Biotinylated MM3 diluted 1/10,000 and SA-PolyHRP diluted 1/8,000 in PBS-T-BSA were subsequently added and incubated for short incubations, after the corresponding washing steps. Finally, OPD (model B) or TMB (model C) substrates were added and after incubation for respectively 20 and 5 min, the reactions were stopped as indicated above and the plates were read at respectively 492 nm and 450 nm.

#### ELISA model D

This method was performed with the same reagents as model A but short incubations of 30 min at RT with shaking at 750 rpm were applied. However, the HRP-labeled goat anti-mouse IgG antibodies used corresponded to a new batch of antibodies from the same commercial source (current batch), which exhibited poorer performance than the former batch used in model A.

### Statistical analysis

The limit of detection for each MM3-COPRO ELISA performed with different secondary reagents and incubation conditions was calculated by testing serial dilutions of known amounts of *F*. *hepatica* ESAs in CoproGuard or in fecal supernatants from pooled negative samples prepared in distilled water. The concentration obtained at the intersection point of the standard curve with the respective cut-off value of each MM3-COPRO ELISA was considered the limit of detection of the assay. The concentrations of the ESAs assayed ranged from 0.15 to 20 ng/mL.

The cut-offs for cattle and sheep were determined for each ELISA model on the basis of values obtained for fecal supernatants from animals not infected with *F*. *hepatica*. Specifically, the cut-offs were determined as being 1 standard deviation (SD) above the highest OD value observed on testing the negative samples from fluke-free cattle and sheep populations. Values higher than this cut-off were considered positive for *F*. *hepatica* infection. This particular method of calculating the cut-off [26] was preferred over other commonly used arbitrary methods based on a statistical parameter such as 2–4 SD above the mean value of negative samples [9, 27]. This ensured maximal specificity while preventing the penalizing effect of the SD parameter when combining a high number of OD values that are close to zero with few data with higher values among negative samples.

The calculated cut-off values were 0.059 (cattle) for ELISA model A (HRP-labeled goat anti-mouse IgG antibodies, former batch), 0.055 (cattle) and 0.064 (sheep) for ELISA model B (SA-PolyHRP and OPD), 0.084 (cattle) and 0.065 (sheep) for ELISA model C (SA-PolyHRP and TMB) and 0.224 (cattle) for ELISA model D (HRP-labeled goat anti-mouse IgG antibodies, current batch). As the secondary NA-HRP reagent was only used for comparison in preliminary ELISA experiments, a specific cut-off was not calculated. Instead, the same cut-off value of SA-PolyHRP was considered as a reference.

Linear correlation coefficients were used to test the relationship between parasite burden and OD values measured by the MM3-COPRO test and between different MM3-COPRO models. The analysis was implemented using the GradPad Instat statistical package (GraphPad Software Inc, CA, USA).

## Results

### Optimizing ELISA conditions for the use of SA-PolyHRP as a secondary reagent for the MM3-COPRO test

**Fig 1:**
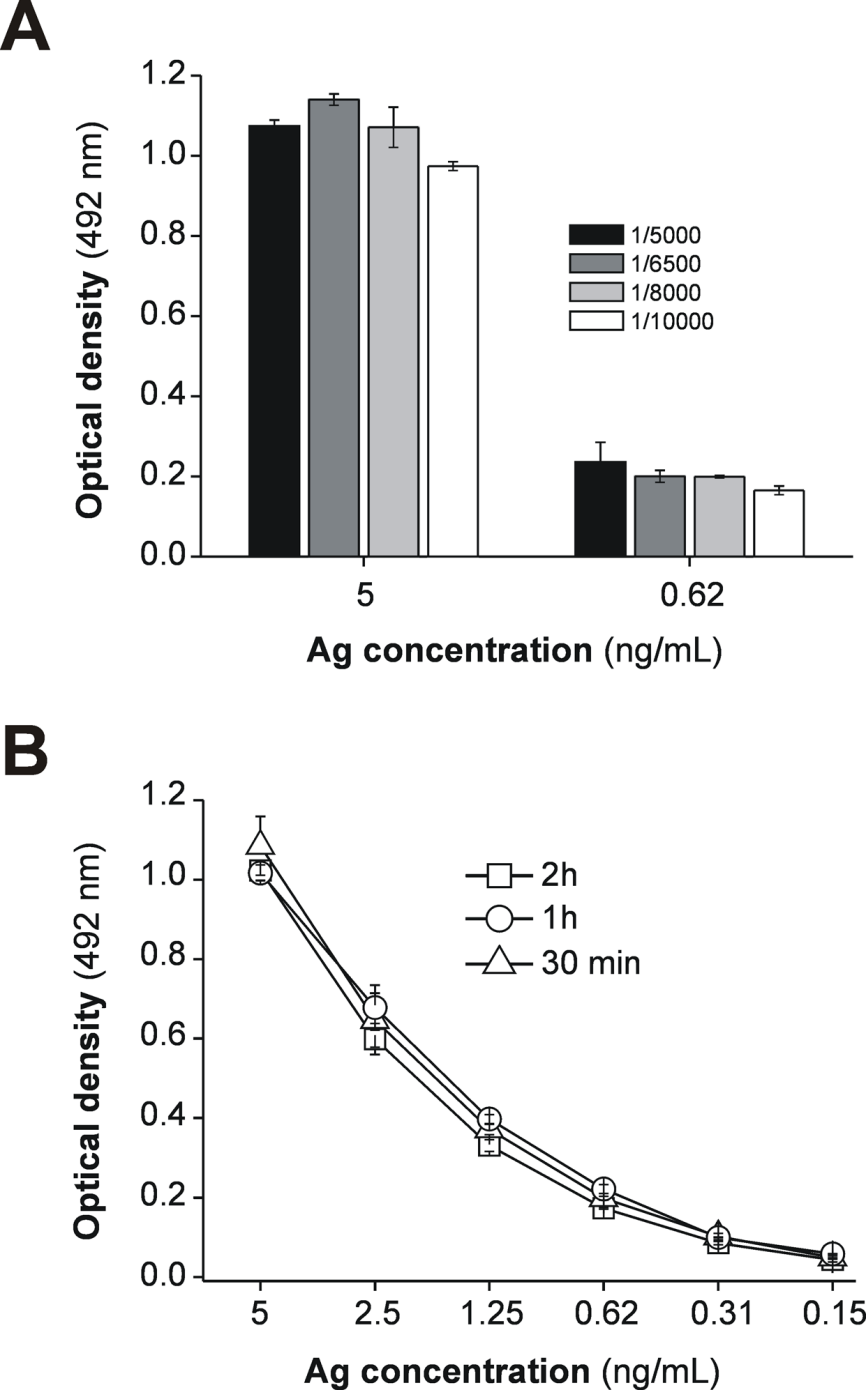
Adaptation of the SA-PolyHRP conjugate and rapid incubations to the MM3-COPRO ELISA. (A) Two concentrations (5 and 0.62 ng/mL) of whole *Fasciola hepatica* excretory-secretory antigens (Ag) diluted in CoproGuard were tested with the MM3-COPRO ELISA by using several dilutions of SA-PolyHRP (ranging from 1/10,000 to 1/5,000) as secondary reagent to reveal bound biotinylated mAb MM3. All incubation steps were performed at RT for 30 min with shaking (750 rpm). (B) Two-fold dilutions (0.15–5 ng/mL) of Ag diluted in CoproGuard were assayed with the MM3-COPRO ELISA (SA-PolyHRP diluted 1/8,000) at RT with shaking for several incubation times (30 min, 1 h and 2 h).

In order to evaluate the usefulness of SA-PolyHRP as a secondary reagent for quantitation of *Fasciola* cathepsins in fecal samples, we first determined the optimal dilution of this reagent and incubation time for samples. The optimization procedure was carried out at RT under shaking and considering several concentrations of antigen diluted in CoproGuard. The data presented in Fig 1A show that the commercial SA-PolyHRP conjugate can be optimally used in the range of 1/5,000-1/10,000 dilution for either medium (5 ng/mL) or low-range (0.62 ng/mL) concentrations of antigen. The data in Fig 1B also indicate that a reduction in the incubation time from 2 h to 30 min under orbital shaking does not decrease the sensitivity of the assay for the overall range of antigen concentrations tested (5 to 0.15 ng/mL), even when incubations were carried out at RT. According to the data obtained, we selected a 1/8,000 dilution and incubations of 30 min at RT with shaking for subsequent comparative experiments.

### The sensitivity of the MM3-COPRO ELISA can be improved using SA-PolyHRP

**Fig 2:**
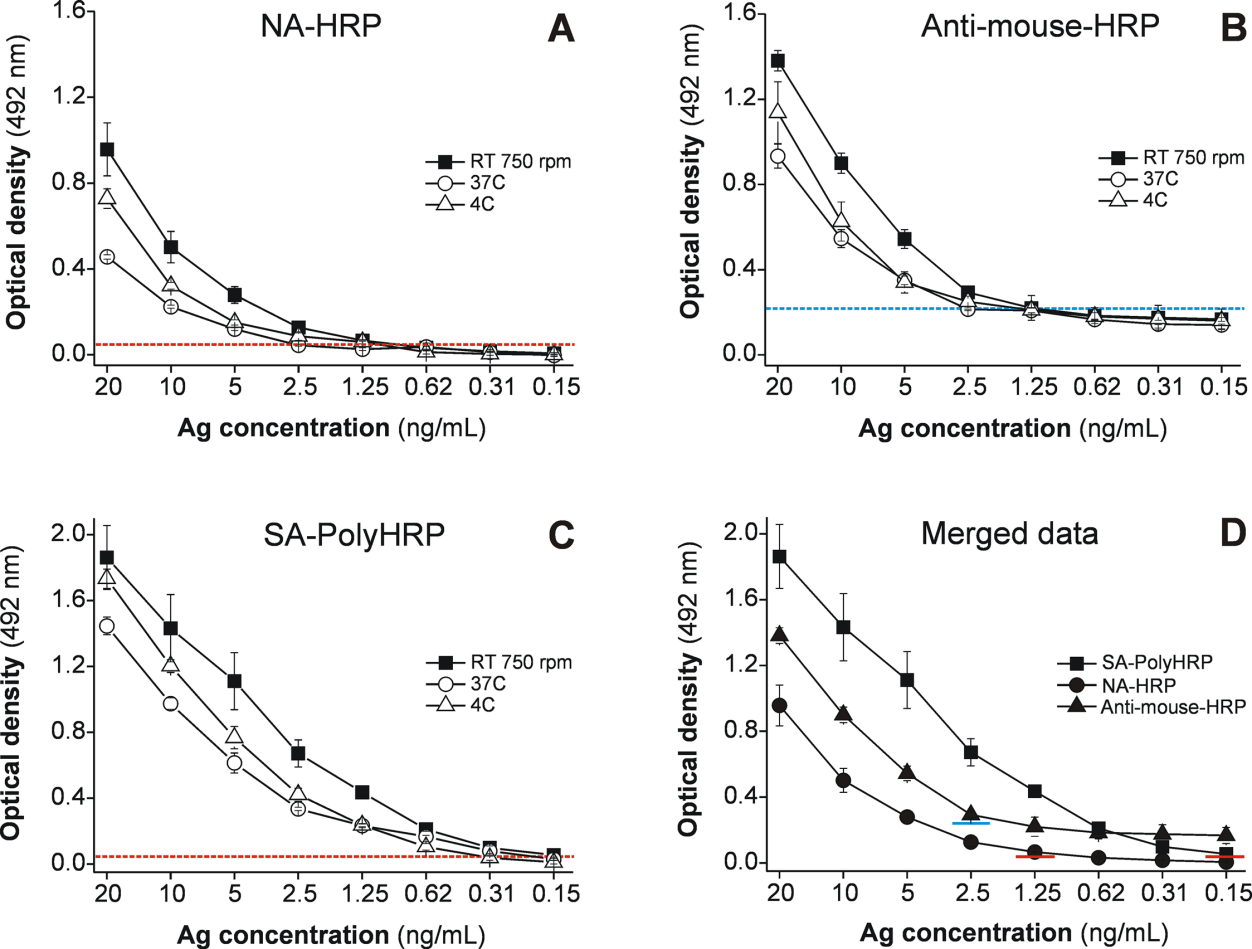
Comparison of three HRP-labeled secondary reagents and several incubation conditions in MM3-COPRO ELISA. The detectability of the MM3-COPRO ELISA was determined by testing two-fold dilutions (0.15–20 ng/mL) of *Fasciola* excretory-secretory antigens (Ag) diluted in CoproGuard, and bound mAb MM3 was revealed with (A) NA-HRP, (B) HRP-labeled anti-mouse IgG antibodies (anti-mouse-HRP) or (C) SA-PolyHRP. The following incubation conditions were considered: 37°C (2 h for samples; 90 min and 1 h for secondary reagents; open circles); 4°C (ON) for incubation of samples and 37°C (90 min and 1 h) for subsequent steps (open triangles); and RT (30 min) with shaking at 750 rpm for all incubation steps (closed squares). (D) Combined data obtained with the three secondary reagents and short incubations at RT (30 min, 750 rpm). The colored lines show the cut-off values calculated for anti-mouse-HRP (OD = 0.224; blue line) and SA-PolyHRP (OD = 0.055; red line) by using negative samples from cattle.

**Fig 3:**
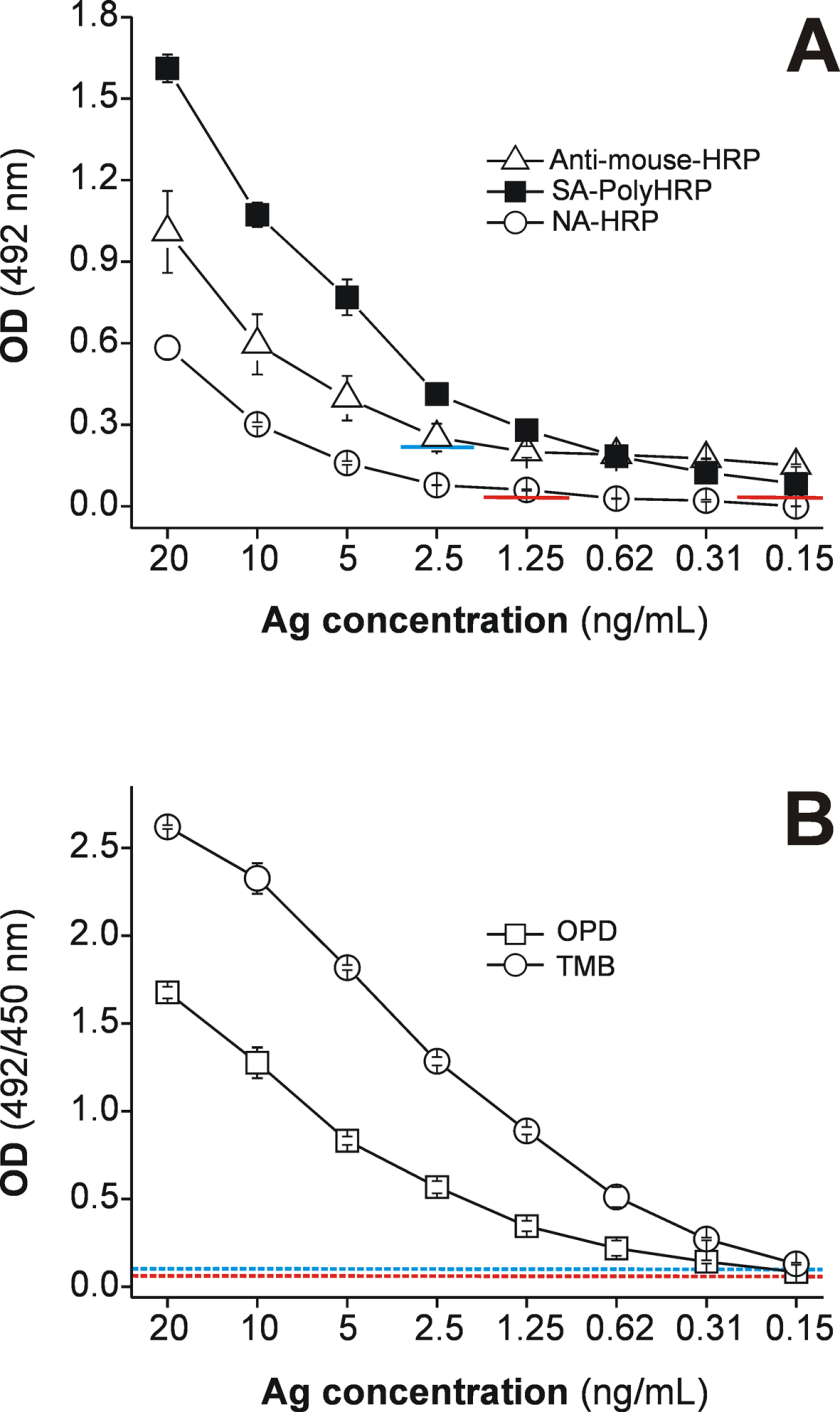
Detectability of rapid MM3-COPRO ELISA performed with three HRP-labeled secondary reagents and two HRP substrates. Two-fold dilutions (0.15–20 ng/mL) of *Fasciola hepatica* excretory-secretory antigens (Ag) diluted in fecal supernatants obtained from pooled negative samples from cattle (prepared with distilled water) were tested under short incubations (30 min, 750 rpm) at RT with (A) HRP-labeled anti-mouse IgG antibodies (triangles; cut-off OD = 0.224, blue line), SA-PolyHRP (squares; cut-off OD = 0.055, red line) and NA-HRP (circles), revealed with OPD substrate; and (B) SA-PolyHRP revealed with two HRP substrates, OPD (squares; cut-off OD = 0.055, dashed red line) and TMB (circles; cut-off OD = 0.084, dashed blue line).

To investigate whether the sensitivity of previous versions of the MM3-COPRO ELISA test can be improved using the SA-PolyHRP system, we compared the OD values obtained with three secondary detection reagents (NA-HRP, HRP-labeled anti-mouse IgG antibodies and SA-PolyHRP), and three incubation conditions: i) 37°C (2 h for samples; 90 min and 1 h for secondary reagents) for all incubation steps, ii) 4°C (ON) for incubation of samples, and 37°C (90 min and 1 h) for subsequent steps, and iii) RT (30 min) with shaking at 750 rpm for all incubation steps. For these experiments, we tested several concentrations of *Fasciola* ESAs diluted in CoproGuard. Comparing the three detection methods, the data in Fig 2A–2D show that SA-PolyHRP provides an advantage over classic HRP-labeled anti-mouse IgG antibodies and NA-HRP detection systems and that incubation for a short period at RT under shaking yields better OD values than longer incubations at 37°C or at 4°C. In particular, for short incubations with agitation (Fig 2D), we observed that the use of SA-PolyHRP in the MM3-COPRO test provided a limit of detection (150 pg/mL) that is about 8 and 16 times lower than that obtained using NA-HRP (1.25 ng/mL) and HRP labeled anti-mouse IgG antibodies (2.5 ng/mL), respectively. When pooled supernatants from negative fecal samples (cattle) extracted with distilled water were used to dilute *Fasciola* ESAs, the OD values for the different antigen concentrations were slightly lower (Fig 3A) than those obtained for the antigen diluted in CoproGuard (Fig 2D); however, the detection limits remained the same for the three secondary reagents tested. Moreover, we observed that the current batch of HRP-labeled anti-mouse antibodies used to perform these experiments yielded a higher background with negative samples in wells containing anti-*Fasciola* antibodies than in control wells with irrelevant antibodies, which was not previously observed with former batches. This effect, which occurred with CoproGuard and with fecal supernatants diluted in distilled water, explains why the OD values of the standard curve obtained with this secondary reagent did not fall below ∽0.200 at concentrations lower than the limit of detection (2.5 ng/ml) (Figs 2C, 2D and 3A).

In addition to the use of enhanced detection systems, ELISA OD values can also be increased by using certain substrates for peroxidase, among which OPD and TMB are frequently used. Comparative results for these two peroxidase substrates are presented in Fig 3B, which shows that the OD values were higher, as expected, with TMB; however, the detectability of the assay (i.e., the smallest amount of analyte detected) [28] did not change as signals in control wells were also higher with TMB.

### Enhancement of MM3-COPRO ELISA signals with SA-PolyHRP did not alter the specificity but increased the sensitivity of the test

**Fig 4:**
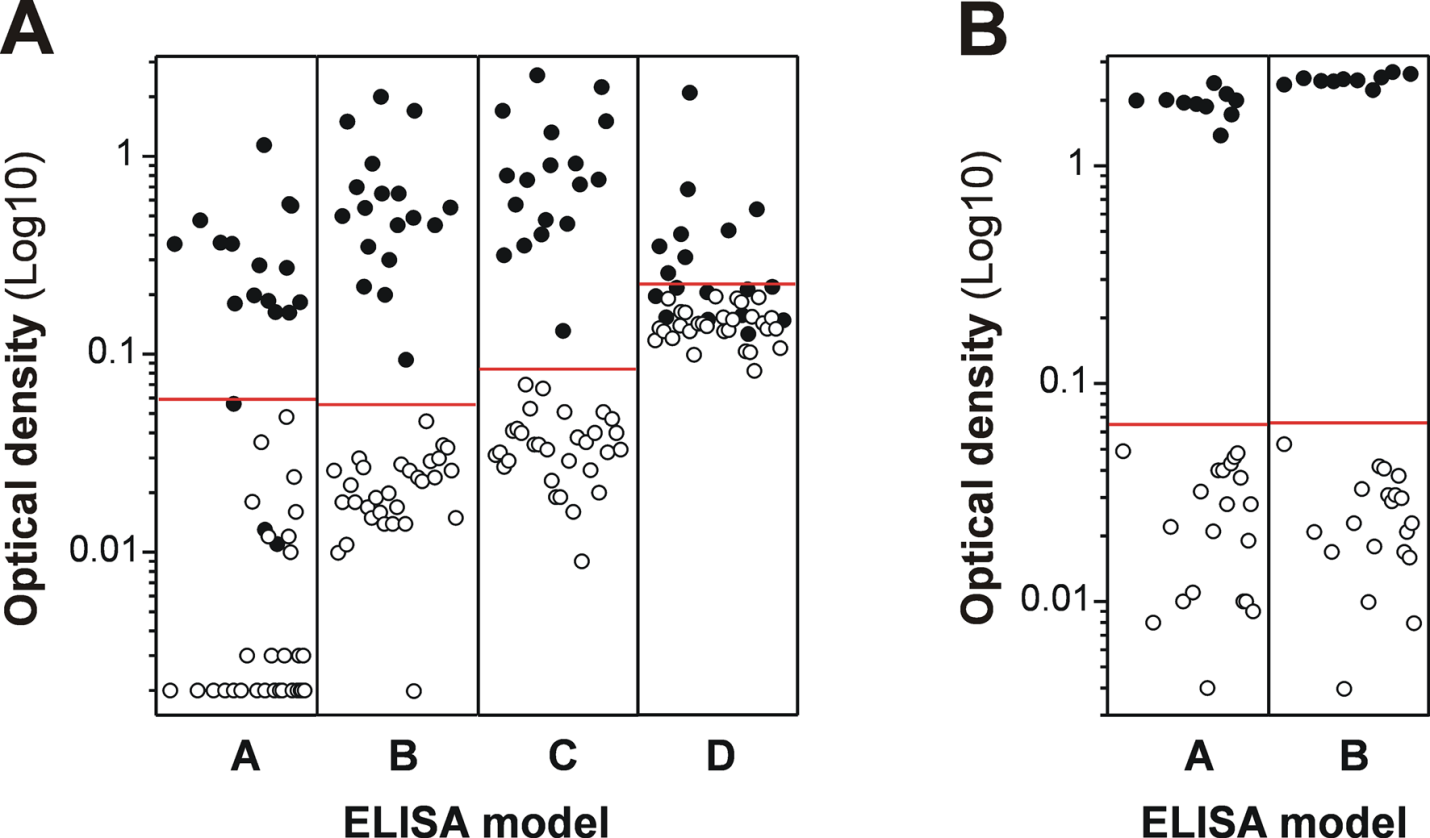
MM3-COPRO OD values obtained for individual *Fasciola hepatica* positive and negative cattle and sheep feces. (A) *F*. *hepatica* positive (n = 18, closed circles) and negative (n = 30, open circles) samples from cows tested with several variations of the MM3-COPRO test (models A-D). (B) *F*. *hepatica* positive (n = 10, closed circles) and negative (n = 20, open circles) samples from sheep tested with models B and C. Model A: classic MM3-COPRO test, performed with standard incubations and a former batch of HRP-labeled anti-mouse IgG antibodies. Model B: performed with short incubations (30 min at RT with shaking), using SA-PolyHRP and OPD. Model C: same as model B, using TMB as substrate. Model D: performed with short incubations and a current batch of HRP-labeled anti-mouse IgG antibodies. The red lines correspond to the cut-off value of each ELISA model: 0.059 for model A (cattle); 0.055 (cattle) and 0.064 (sheep) for model B; 0.084 (cattle) and 0.065 (sheep) for model C; and 0.224 (cattle) for model D.

In order to determine the sensitivity and specificity of the MM3-COPRO test by using the SA-PolyHRP secondary reagent and to obtain a cut-off value, we evaluated its ability to correctly classify positive and negative bovine and ovine fecal samples. Two peroxidase substrates, OPD and TMB, were also evaluated. The data in Fig 4 show the results obtained with positive and negative samples with the classic MM3-COPRO ELISA (model A, using a former batch of HRP-labeled goat anti-mouse IgG polyclonal antibodies and performed at the time of sample collection), the enhanced MM3-COPRO revealed with SA-PolyHRP and OPD (model B) or TMB (model C), and the MM3-COPRO performed using a current batch of goat anti-mouse IgG-HRP conjugate (model D). As indicated in the previous section, negative samples (open circles) from cattle (Fig 4A) and sheep (Fig 4B) were used to calculate the cut-off value for each model. Regarding the positive bovine samples (Fig 4A, closed circles), models B and C were able to correctly classify all samples and the signal-to-noise ratios were optimal for both. Nevertheless, as expected, higher OD values and cut-off values were obtained when SA-PolyHRP secondary reagent and the TMB substrate were combined (model C, cut-off value = 0.084) than with the OPD substrate (model B, cut-off value = 0.055). Unlike the enhanced MM3-COPRO methods (models B and C), models A and D yielded poorer results with multiple mismatches. In particular, the accuracy of these models was highly dependent on the performance of each batch of polyclonal antibodies. Overall, the results obtained with model A were acceptable, as of the three false negative samples, two corresponded to animals harboring a single fluke and the other corresponded to an animal harboring 4 flukes but with an OD signal (OD = 0.056) close to the cut-off value (OD = 0.059). However, with model D, the results were diagnostically unacceptable, as the cut-off value was very high (OD = 0.224) and consequently 10/18 positive samples (55.6%) were incorrectly classified. Positive fecal samples from experimentally infected sheep were also tested with enhanced models B and C (Fig 4B, closed circles). All samples produced a high response and no significant differences were observed on comparing the OD values from animals harboring low fluke burdens (2–5 flukes, 6 animals) and those harboring medium to high fluke burdens (12–28 flukes, 4 animals). Model A was also used to analyze sheep samples at the time of sample collection and also correctly classified the samples.

### Correlation between MM3-COPRO models and fluke burden, and kinetics of coproantigen release

**Fig 5:**
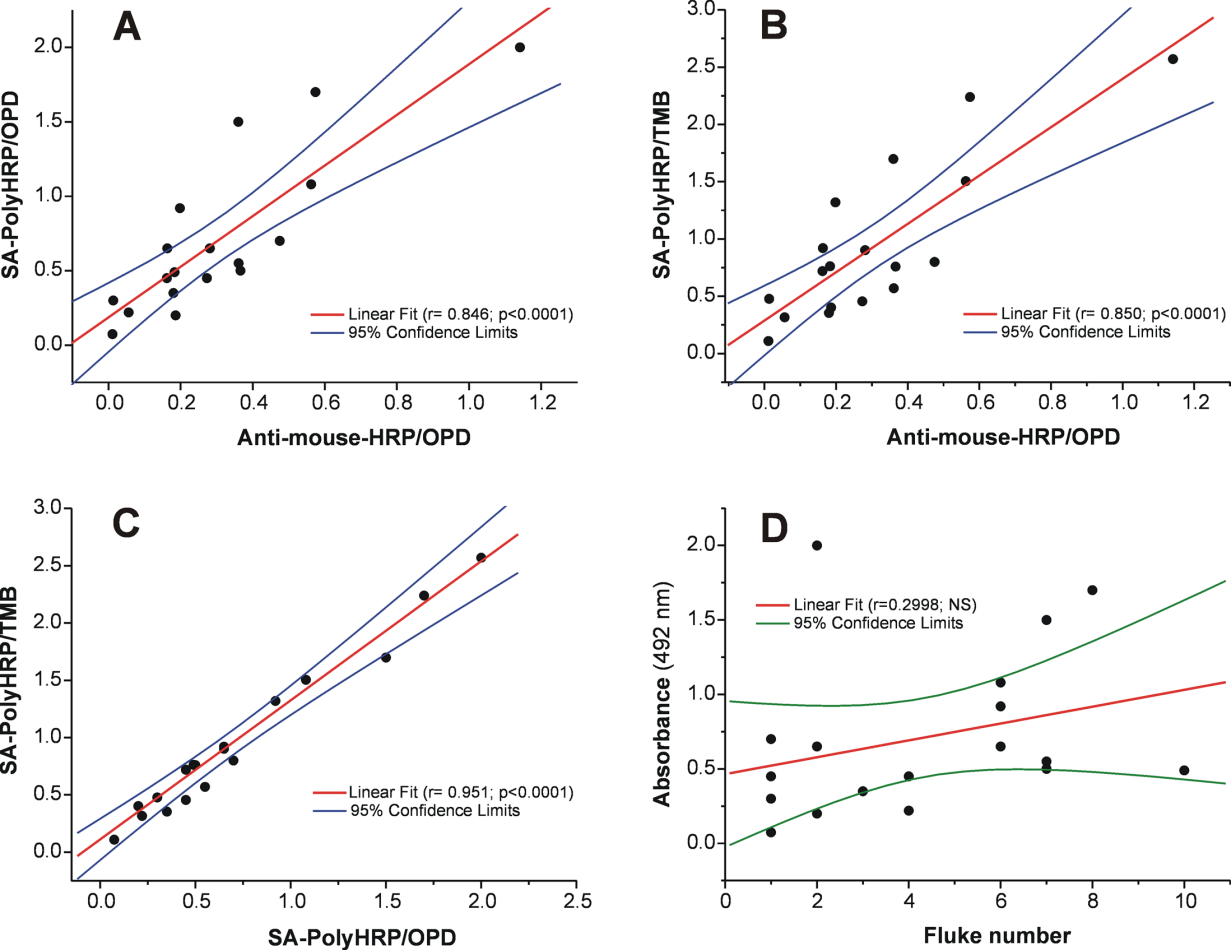
Correlation between MM3-COPRO models and fluke burden. Each point represents an individual cow naturally infected with *Fasciola hepatica* (n = 18) and tested with several variations of the MM3-COPRO test. (A) Plot of the OD values measured by MM3-COPRO model B (SA-PolyHRP and OPD, rapid incubations) against model A (HRP-labeled anti-mouse IgG antibodies, former batch, and OPD, standard incubations). (B) Plot of the OD values obtained with model C (SA-PolyHRP and TMB, rapid incubations) against model A. (C) Plot of the OD values measured by model C against model B. (D) Plot of the OD values determined by model B against the number of flukes recovered at necropsy.

**Fig 6:**
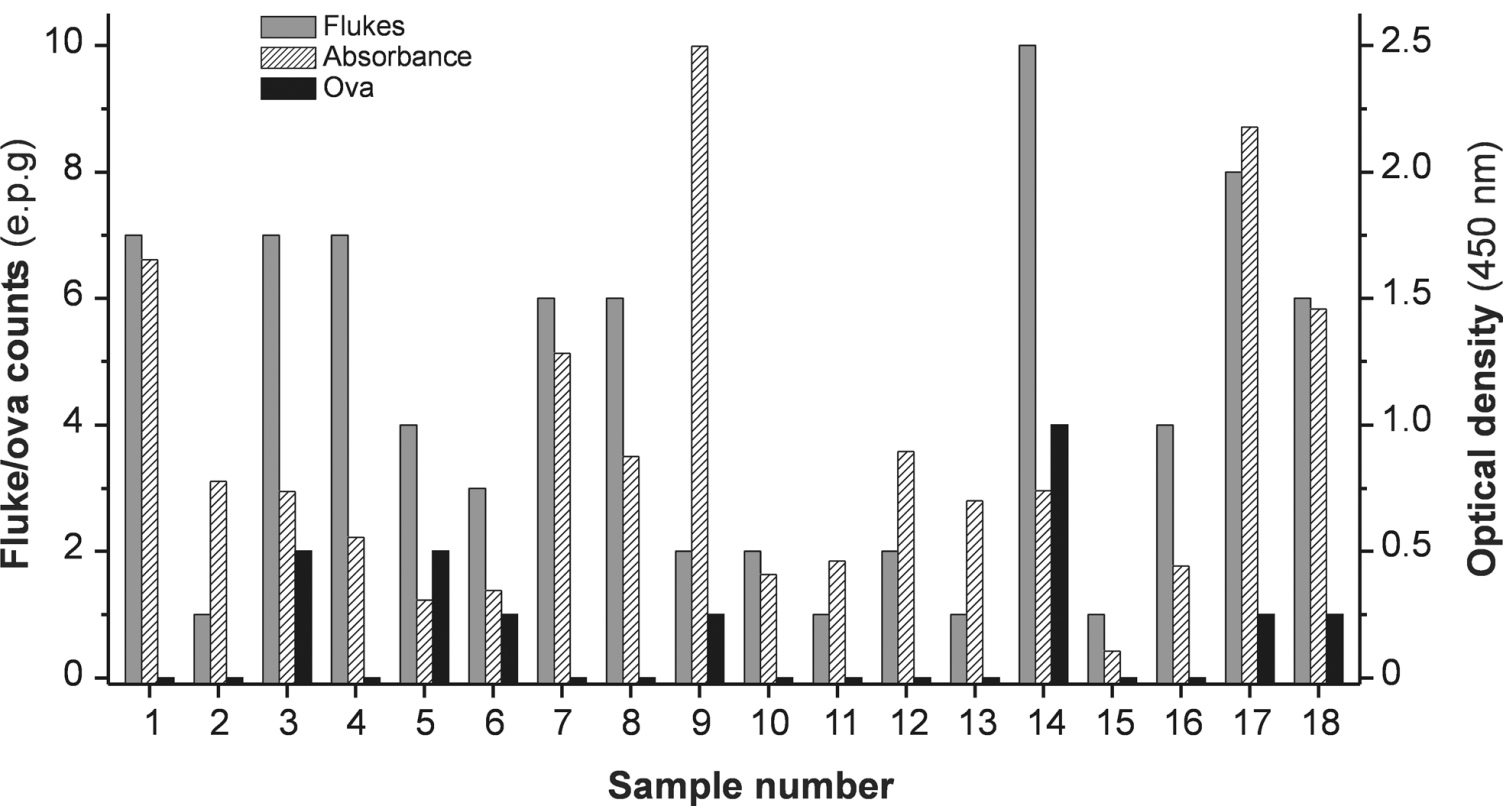
Fecal egg counts, fluke burden and OD values obtained by testing fecal samples from cows naturally infected with *Fasciola hepatica* (n = 18) with the rapid enhanced MM3-COPRO ELISA. Fluke burdens were determined at necropsy. MM3-COPRO ELISA was performed with short incubations (30 min at RT with shaking) and with SA-PolyHRP as secondary reagent, which was revealed with TMB (model C).

Considering samples from cattle naturally infected with *F*. *hepatica* (n = 18), a close correlation (Fig 5A and 5B) was observed on comparing the classic MM3-COPRO ELISA (model A) with both enhanced MM3-COPRO ELISAs (model B and C; r = 0.846 and r = 0.850, respectively; *p*<0.0001). As expected, the correlation coefficient was even higher when models B and C were compared (Fig 5C; r = 0.951; *p*<0.0001). However, we did not observe a correlation between fluke burden in liver and the OD values obtained with the MM3-COPRO ELISA, as shown for the ELISA model B in Fig 5D (r = 0.2998; *p* = 0.2267). As additional data, the results in Fig 6 show the number of parasites collected from the liver, e.p.g. counts in feces, and the ELISA OD values (model C) obtained for each animal. As expected, most samples (11/18) were negative for the presence of eggs by microscopic examination. Among positive samples, the presence of *Fasciola* eggs was low (ranging from 1 to 4 e.p.g., the latter corresponding to the only animal harboring 10 flukes; sample #14).

**Fig 7:**
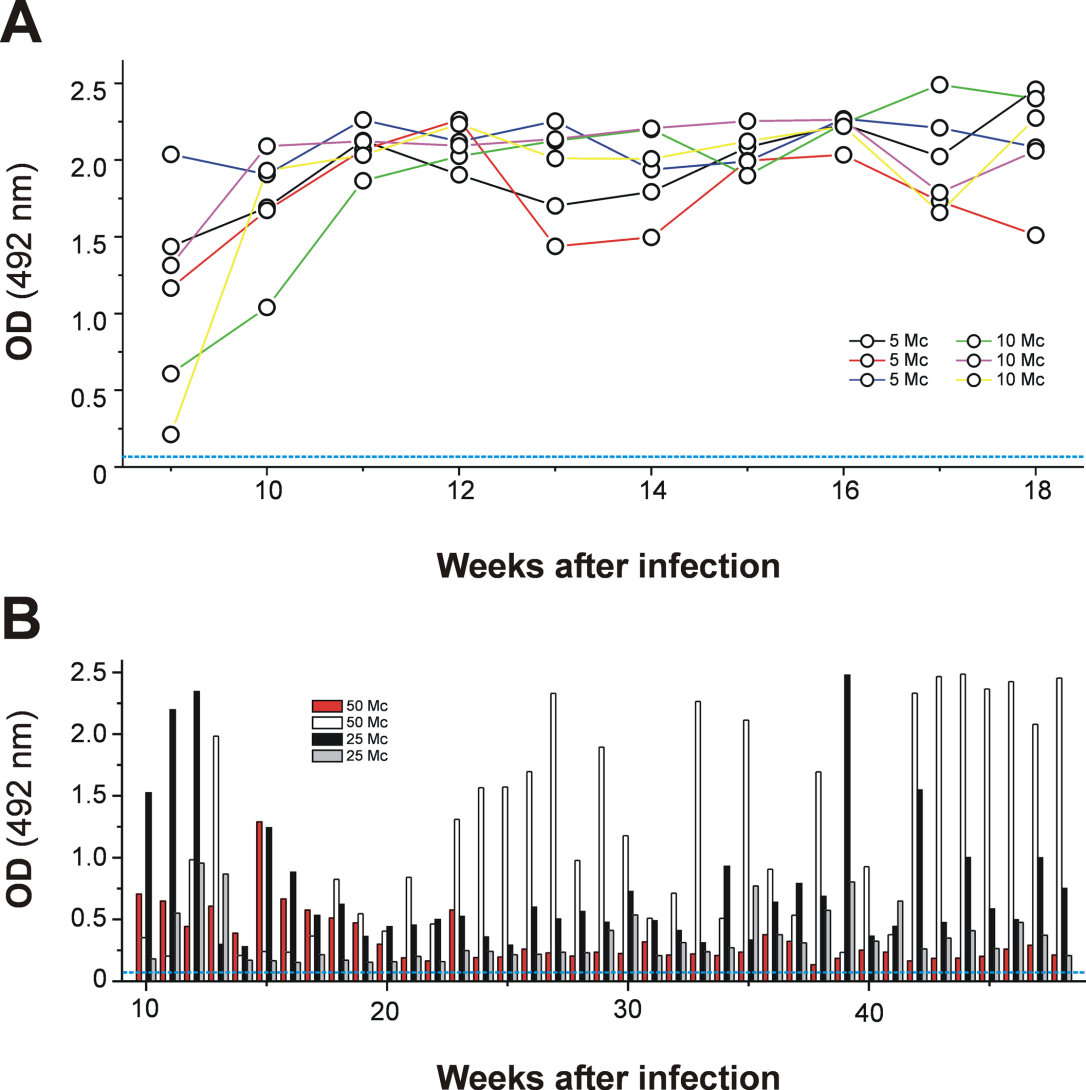
OD values obtained with the rapid enhanced MM3-COPRO ELISA testing fecal samples from animals experimentally infected with *Fasciola hepatica*. (A) Lambs were experimentally infected with 5 (n = 3) or 10 (n = 3) metacercariae. (B) Cows were infected with 25 (n = 2) and 50 (n = 2) metacercariae (Mc). Fecal samples were obtained weekly from each animal during the period of study and analyzed with the MM3-COPRO test using SA-PolyHRP and OPD and short incubations (model B). The cut-off value is 0.064 for sheep and 0.055 for cattle.

Regarding the sensitivity of the enhanced MM3-COPRO ELISAs, it is also interesting to determine whether the tests can correctly classify positive fecal samples collected at regular intervals during the biliary phase of the parasite from animals infected with a small number of metacercariae, considering the usual fluctuations in antigen concentration in feces. For this purpose, we investigated the kinetics of coproantigen release in fecal samples obtained sequentially (weekly) from lambs and cattle experimentally infected with different doses of *F*. *hepatica* metacercariae during the patent period of infection. As the enhanced MM3-COPRO ELISA performs similarly with OPD and TMB, only the former substrate was considered. The data in Fig 7A show that the release of coproantigen in sheep remained high and stable throughout the period of infection investigated (weeks 9–18). No differences were observed for fecal samples obtained from animals infected with either 5 (fluke recovery of 53%; fluke burdens: 2, 3 and 3) or 10 (fluke recovery of 30%; fluke burdens: 1, 3 and 5) metacercariae. On the contrary, regarding fecal samples from positive cattle, very variable OD values were obtained, although the test was able to correctly classify as positive all samples obtained from each animal (Fig 7B). Again, no differences were observed on comparing samples from animals infected with either 50 or 25 metacercariae. Finally, in order to obtain information about the number of flukes that reached adult stages in the infected cows, one animal infected with 25 metacercariae was sacrificed in the slaughterhouse at the end of the experiment. Only 3 adult flukes were found in liver. Although only one animal was slaughtered, this indicates that the fluke burden in the animals used in this experiment was probably low as in the group of 18 animals selected at slaughterhouse to test the sensitivity of the different ELISA methods.

## Discussion

Detection of minimum amounts of *Fasciola* L-cathepsins in fecal samples by capture ELISA has emerged as one of the best ways of confirming early infections by this trematode in humans and animal species and of monitoring the efficacy of anthelmintic treatments [5, 9]. Moreover, as indicated by Valero *et al*. [29], some of these methods can be used for rapid mass screening, early detection of treatment failure or re-infection in post-treated subjects and are also useful for surveillance programs. In previous studies using our in-house MM3-COPRO ELISA [9], we observed that only a low percentage of fecal samples from cows harboring one fluke (2/7 = 28.6%) tested positive, while all samples from animals parasitized with two or more flukes were correctly classified. Using the same MM3-COPRO ELISA method (model A), we observed that this ELISA was unable to correctly classify 2/4 (50%) of positive fecal samples from animals harboring a single fluke and also that one sample from an animal harboring four flukes tested negative. Moreover, the number of false negative results with samples from animals with a low parasite burden increased substantially on using currently available HRP-conjugated anti-mouse IgG polyclonal antibodies (model D), which prevented assessment of the overall performance of the original MM3-COPRO ELISA (model A). Although the reasons why the results obtained with polyclonal antibodies vary so greatly from batch to batch are not provided by the manufacturing companies, we hypothesize that it may be due to variations in the immunological response of the immunized animals and also to contaminants generated during immunization and/or antibody purification procedures. For example, small amounts of A/G proteins, as well as positively charged polymer fragments, released from either affinity or DEAE columns during antibody purification, may generate high background levels [30]. In addition, purification methods that do not isolate antigen-specific antibodies [31] can copurify anti-IgG antibodies (if present) that might react with immunoglobulins from other species not used for immunization. In particular, the latter situation may represent a strong source of background in ELISAs that combine polyclonal and monoclonal antibodies, for capture and detection steps, such as the MM3-COPRO and MM3-SERO ELISAs.

An alternative to peroxidase-labeled species-specific anti-IgG polyclonal antibodies is the use of mAbs labeled with haptens such as FITC [32] or biotin [10, 33], which can be subsequently recognized by peroxidase-labeled anti-FITC antibodies or peroxidase-labeled NA/SA reagents, respectively. In our study, we first evaluated the performance of the test using MM3-biotin and NA-HRP reagents in order to modify our classic MM3-COPRO ELISA. However, as can be deduced from the results presented in Figs 2D and 3A, although the use of NA-HRP to detect *Fasciola* L-cathepsins in fecal samples is highly specific and generates low background levels, the ELISA detection ability was only slightly better than that obtained with the current batch of HRP-labeled anti-mouse IgG polyclonal antibodies. Nevertheless, the performance of the MM3-COPRO ELISA could be significantly improved by using biotinylated MM3 and SA-PolyHRP as the amplification system. Regular NA-HRP and SA-HRP conjugates are among the most frequently used immune detection molecules in ELISA for general application. However, when detection of small amounts of analyte in samples is required, enhancement systems that exploit the use of recognition molecules containing a large proportion of enzymes may be required. Reagents based on this principle can be produced by the attachment of the recognition molecules of interest and multiple enzyme molecules to a backbone of dextran [34, 35] or by conjugation of polymeric HRP to multiple recognition molecules such as SA [30].

Among the available enhancement systems, we decided to use SA-PolyHRP conjugates for four main reasons: i) the coupling of biotin to mAb MM3 has previously been standardized [10] and is already used in the commercial version of the kit (BIO K 201, BIO X Diagnostics); ii) SA-PolyHRP is a versatile reagent available in different homopolymer lengths and from several commercial sources; iii) it can directly substitute SA/NA-HRP in biotin-SA/NA based ELISAs with minimal adjustments; and iv) although biotin is present in feces, as it is present in food and synthesized by intestinal flora [36], it does not interfere in our MM3-COPRO ELISA because it is removed during subsequent washings before adding the SA-PolyHRP conjugate.

As noted in the previous section, the enhancement of MM3-COPRO ELISA values with SA-PolyHRP enabled us to correctly classify all positive and negative samples, yielding an excellent signal-to-noise ratio and a detection limit of 150 pg/mL; this low limit has not previously been achieved with methods of detecting *Fasciola* coproantigens. Moreover, considering that the standard curve constructed for calibrating the assay uses the total protein content in *Fasciola* ESAs and that L-cathepsins, although the most abundant [37], are not the only proteins present, the true detection limit of the assay is expected to be even lower than 150 pg/mL.

ELISA methods that can detect very small amounts of *Fasciola* coproantigens are of great interest, particularly given the high day-to-day variability in the antigen concentration in fecal samples from large ruminants. In this sense, 2–6 fold variations in antigen concentration throughout consecutive daily collections from cows infected with a medium to large number of adult flukes in liver has already been reported [11]. Similarly, in the present study we observed a high variability in coproantigen release in fecal samples collected weekly from cattle experimentally infected with 25 and 50 metacercariae (Fig 7B). In addition, there were large differences (6–12 fold) in OD values of single samples obtained from cattle harboring only 1–10 flukes (Fig 4A). Considering fluke burdens, we also observed that OD differences were higher, as expected, for fecal samples from cattle harboring a single adult fluke (OD range = 0.094–0.7; model C) than for cattle parasitized with 6–10 flukes (OD range = 0.5–1.7; model C). This may explain the absence of correlation between fluke burden and the OD values observed in the present study (Fig 5D).

For animals with the lowest fluke burden (e.g. 1–4 parasites), we hypothesize that differences in the release of L-cathepsins in feces may be due to a different size/maturation status of the flukes or due to the elapsed time between cyclic regurgitations. However, although the high variability in antigen concentration in cows sampled daily/weekly was proposed to be related either to biological effects of the parasite, the effects of developing pathology and immunity, or changes in fecal consistency [11], such a high variation in cows with a high number of parasites was never convincingly explained.

Unlike for the cattle samples, the results obtained with MM3-COPRO ELISA models B and C testing sheep samples collected at 13 wpi showed similar high OD values independently of fluke burden (2–28 flukes; Fig 4B). Fluctuations in antigen were less apparent in fecal samples obtained weekly from sheep experimentally infected with 5 or 10 metacercariae (Fig 7A). This can be explained by the fact that with the enhanced MM3-COPRO ELISA, the concentration of antigen in feces is high enough to yield saturated OD values, even when variations in antigen concentration occur. In agreement with this, in a study by Mezo et al. [9], coproantigen concentrations in experimentally infected lambs harboring 1–36 flukes were in all cases over 12 ng/mL, which would result in OD values higher than 1.0 with the enhanced MM3-COPRO ELISA.

The observation that the OD values obtained with samples from animals with one or two flukes may be higher than that of animals harboring, for example, 10 flukes (Fig 6), underlines the importance of using highly sensitive ELISA methods as the proposed enhanced MM3-COPRO ELISA. It could be argued that such sensitivity is not necessary, as it has been reported that only animals harboring 10 or more flukes suffer significant hepatic damage [12]. However, highly sensitive methods are required in at least three situations: i) poorly sensitive methods cannot guarantee the detection of a positive animal by testing a single fecal sample due to the high variability in L-cathepsins concentration in feces; ii) ultra-sensitive methods are particularly useful for detecting the survival of flukes after anthelmintic treatment due to resistance, which is of great interest to prevent spreading resistant strains; and iii) detection of animals with low fluke burdens may be of additional interest as it has been reported that *Fasciola* infections promote TH2 responses, which in turn may favor infections by certain pathogens and even confound the diagnosis of diseases such as bovine tuberculosis, caused by *Mycobacterium bovis* [38]. Moreover, highly sensitive methods are essential for early diagnosis of human infections by *F*. *hepatica* or *F*. *gigantica*.

In addition to coproantigen determinations in fecal samples, the presence of fluke infections within dairy herds can also be investigated by examining the presence of antibodies in serum or in bulk tanks [6, 39]. However, for beef herds, composite fecal egg counts mixing feces from up to 10 animals and further investigation of animals that contribute to the positive samples are frequently used in sedimentation assays [40]. Pooled fecal samples have also been used with the coproantigen ELISA in some studies [11, 13]. Although we cannot recommend a priori analysis of coproantigen in composite fecal samples, as this practice can misdiagnose positive animals particularly in days coincident with low coproantigen elimination, the improved sensitivity of the enhanced MM3-COPRO reported in this study warrants further investigation of this possibility.

Finally, as mAb MM3 also recognizes L-cathepsins from *F*. *gigantica* [41, 42] and the method was already used for detection of *F*. *gigantica* coproantigens in feces of sheep experimentally infected with this species [43], it is expected that the ELISA modifications proposed here are also applicable in regions where only *F*. *gigantica* or both species are present. However, as the consistency and alimentation of animals may differ from one region to another, and the polyclonal antibodies used to capture antigens in the MM3-COPRO ELISA were produced using ESAs from *F*. *hepatica*, adaptation of cut-off values and/or substitution of the polyclonal antibodies by others obtained by immunizing rabbits with ESAs from *F*. *gigantica* may be necessary.

In summary, the MM3-COPRO ELISA is improved by incorporating the SA-PolyHRP as a detection system, thus yielding a robust method that can detect very low levels of antigen in feces from animals infected with *Fasciola hepatica* and that enables detection of infection in cattle parasitized with a single fluke. Moreover, this method was able to correctly classify positive animals throughout the patent period considered in experimental infections. This may solve sensitivity-related problems caused by the broad daily antigen oscillations observed in large ruminants. As the classic MM3-COPRO ELISA has already been tested with human fecal samples [10, 29] the proposed modification is also expected to be useful for improving the early diagnosis of human infections by *Fasciola* species after adjusting the cut-off value. Preliminary studies being carried out in our laboratory with fecal human samples seem to confirm this expectation.

### Limitations

The limitations of the study lay in the small sample size, which was conditioned by the availability of eligible samples and the complexity of performing multiple simultaneous test comparisons. Nevertheless, we estimate that the number of samples used was adequate to draw robust conclusions about the better performance of the MM3-COPRO test using the SA-PolyHRP conjugate with respect to the other secondary reagents evaluated, which was the main purpose of this study. This does not preclude that future studies conducted with a large number of samples from areas of different disease prevalence, feeding and farming conditions are required to obtain definitive conclusions about the performance of the proposed methodology.

## Supporting Information

S1 Fig STARD flowchart of the study. (PPTX)

S1 Table STARD checklist of the study. (DOCX)
