# Supplementary material for: Rapid Enhanced MM3-COPRO ELISA for Detection of Fasciola Coproantigens
Source: PLoS Negl Trop Dis. 2016 Jul 20;10(7):e0004872. doi: 10.1371/journal.pntd.0004872 (PMC4954672; doi:10.1371/journal.pntd.0004872)
Supplement: S1 Fig — (PPTX) [file pntd.0004872.s001.pptx]

## Slide 1
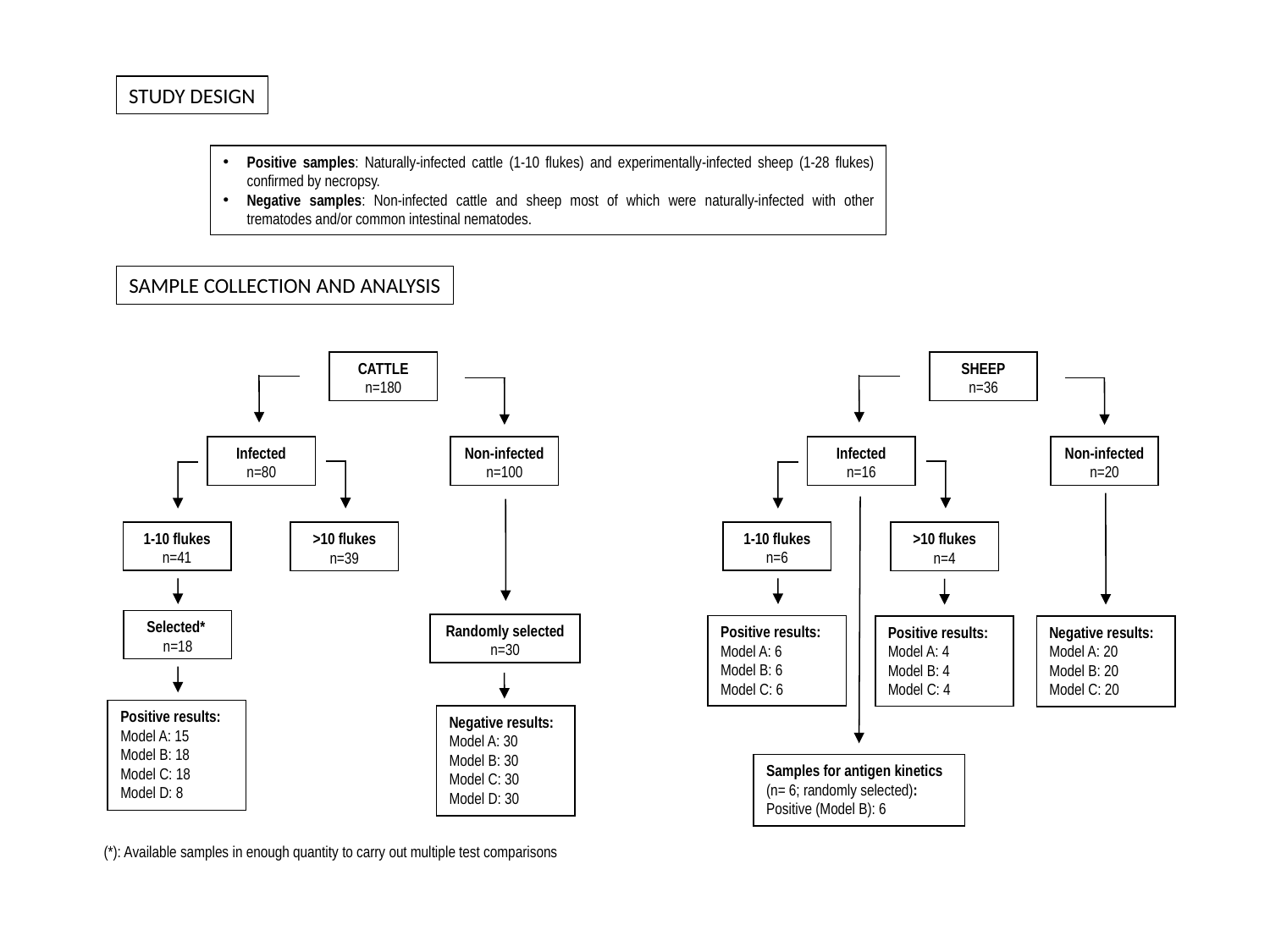

STUDY DESIGN
Positive samples: Naturally-infected cattle (1-10 flukes) and experimentally-infected sheep (1-28 flukes) confirmed by necropsy.
Negative samples: Non-infected cattle and sheep most of which were naturally-infected with other trematodes and/or common intestinal nematodes.
SAMPLE COLLECTION AND ANALYSIS
SHEEP
n=36
CATTLE
n=180
Non-infected
n=100
Infected
n=80
1-10 flukes
n=41
>10 flukes
n=39
Selected*
n=18
Randomly selected
n=30
Positive results:
Model A: 15
Model B: 18
Model C: 18
Model D: 8
Negative results:
Model A: 30
Model B: 30
Model C: 30
Model D: 30
Non-infected
n=20
Infected
n=16
1-10 flukes
n=6
>10 flukes
n=4
Positive results:
Model A: 6
Model B: 6
Model C: 6
Positive results:
Model A: 4
Model B: 4
Model C: 4
Negative results:
Model A: 20
Model B: 20
Model C: 20
Samples for antigen kinetics (n= 6; randomly selected):
Positive (Model B): 6
(*): Available samples in enough quantity to carry out multiple test comparisons
