# Supplementary material for: Rapid Enhanced MM3-COPRO ELISA for Detection of Fasciola Coproantigens
Source: PLoS Negl Trop Dis. 2016 Jul 20;10(7):e0004872. doi: 10.1371/journal.pntd.0004872 (PMC4954672; doi:10.1371/journal.pntd.0004872)
Supplement: S1 Table — (DOCX) [file pntd.0004872.s002.docx]

Table S1. STARD checklist for reporting of studies of diagnostic accuracy

| **Section & Topic** | **No** | **Item** | **Reported on page #** |
| --- | --- | --- | --- |
| **TITLE OR ABSTRACT** | 1 | Identification as a study of diagnostic accuracy using at least one measure of accuracy (such as sensitivity, specificity, predictive values, or AUC) | 1 and 2 |
| **ABSTRACT** | 2 | Structured summary of study design, methods, results, and conclusions  (for specific guidance, see STARD for Abstracts) | 2 |
| **INTRODUCTION** |  |  |  |
|  | 3 | Scientific and clinical background, including the intended use and clinical role of the index test | 4, 5 |
|  | 4 | Study objectives and hypotheses | 5 |
| **METHODS** |  |  |  |
| *Study design* | 5 | Whether data collection was planned before the index test and reference standard  were performed (prospective study) or after (retrospective study) | 6 |
| *Participants* | 6 | Eligibility criteria | 6 |
|  | 7 | On what basis potentially eligible participants were identified  (such as symptoms, results from previous tests, inclusion in registry) | 6 |
|  | 8 | Where and when potentially eligible participants were identified (setting, location and dates) | Not applicable |
|  | 9 | Whether participants formed a consecutive, random or convenience series | 5 |
| *Test methods* | 10a | Index test, in sufficient detail to allow replication | 7, 8, 9 |
|  | 10b | Reference standard, in sufficient detail to allow replication | 6 |
|  | 11 | Rationale for choosing the reference standard (if alternatives exist) | None |
|  | 12a | Definition of and rationale for test positivity cut-offs or result categories  of the index test, distinguishing pre-specified from exploratory | 10 |
|  | 12b | Definition of and rationale for test positivity cut-offs or result categories  of the reference standard, distinguishing pre-specified from exploratory | Not applicable |
|  | 13a | Whether clinical information and reference standard results were available  to the performers/readers of the index test | Not applicable |
|  | 13b | Whether clinical information and index test results were available  to the assessors of the reference standard | Not applicable |
| *Analysis* | 14 | Methods for estimating or comparing measures of diagnostic accuracy | 10 |
|  | 15 | How indeterminate index test or reference standard results were handled | Not applicable |
|  | 16 | How missing data on the index test and reference standard were handled | Not applicable |
|  | 17 | Any analyses of variability in diagnostic accuracy, distinguishing pre-specified from exploratory | Not applicable |
|  | 18 | Intended sample size and how it was determined | Not applicable |
| **RESULTS** |  |  |  |
| *Participants* | 19 | Flow of participants, using a diagram | S1 Figure |
|  | 20 | Baseline demographic and clinical characteristics of participants | Not applicable |
|  | 21a | Distribution of severity of disease in those with the target condition | Not applicable |
|  | 21b | Distribution of alternative diagnoses in those without the target condition | Not applicable |
|  | 22 | Time interval and any clinical interventions between index test and reference standard | Not applicable |
| *Test results* | 23 | Cross tabulation of the index test results (or their distribution)  by the results of the reference standard | Figure 4 and S1 Figure |
|  | 24 | Estimates of diagnostic accuracy and their precision (such as 95% confidence intervals) | Not applicable |
|  | 25 | Any adverse events from performing the index test or the reference standard | Not applicable |
| **DISCUSSION** |  |  |  |
|  | 26 | Study limitations, including sources of potential bias, statistical uncertainty, and generalisability | 20 |
|  | 27 | Implications for practice, including the intended use and clinical role of the index test | 18,19, 20 |
| **OTHER INFORMATION** |  |  |  |
|  | 28 | Registration number and name of registry | Not applicable |
|  | 29 | Where the full study protocol can be accessed | Not applicable |
|  | 30 | Sources of funding and other support; role of funders | Financial disclosure section |
